# *GSTT1* and *GSTM1* polymorphism in cigarette smokers with head and neck squamous cell carcinoma

**DOI:** 10.1016/S1808-8694(15)31022-3

**Published:** 2015-10-19

**Authors:** Joice Matos Biselli, Renata Cristina de Angelo Calsaverini Leal, Mariângela Torreglosa Ruiz, Eny Maria Goloni-Bertollo, José Victor Maníglia, Andréa Regina Baptista Rossit, Érika Cristina Pavarino-Bertelli

**Affiliations:** a Biology graduate, Master's degree graduate student on Health Sciences at the Sao Jose do Rio Preto Medical School - FAMERP.; b Physical therapist, Master's degree graduate student on Health Sciences at the Sao Jose do Rio Preto Medical School - FAMERP.; c Master on Biological Sciences, Doctoral graduate on Health Sciences at the Sao Jose do Rio Preto Medical School - FAMERP.; d Lecturing Professor at the Sao Jose do Rio Preto Medical School - FAMERP Molecular Biology Department.; e Lecturing Professor in the Otorhinolaryngology and Head & Neck Surgery Department at the Sao Jose do Rio Preto Medical School - FAMERP.; f Doctor on Biological Sciences (Genetics), Adjunct Professor of the Infectious and Parasitological Dermatological Diseases Department.; g Dovtor on Biological Sciences (Genetics), Adjunct Professor of the Molecular Biology Department at the Sao Jose do Rio Preto Medical School - FAMERP. Sao Jose do Rio Preto Medical School - FAMERP. Molecular Biology Department - Research Unit on Genetics and Molecular Biology - UPGEM.

**Keywords:** Head and neck neoplasms, Polymorphism, Tobacco, Alcohol, Glutathione transferase

## Abstract

Gene variability related to carcinogen activation and detoxification may interfere with susceptibility to head and neck cancer.

**Aim:**

To investigate the relation between *GSTT1* and *GSTM1* null polymorphisms and the risk of head and neck squamous cell carcinoma in cigarette smokers.

**Material and Method:**

A case-control study conducted at the Sao Jose do Rio Preto Medical School, Brazil. *GSTM1* and *GSTT1* null genotype frequencies were evaluated by multiplex PCR in ^45^ cigarette smokers with head and neck squamous cell carcinomas and ^45^ cigarette smokers without this disease.

**Results:**

The oral cavity was the most prevalent tumor site for squamous cell carcinoma. The *GSTT1* null genotype was found in 33.3% of the Experimental Group and 23.3% of the Control Group (p= 0.311). Experimental and Control Groups had *GSTM1* null genotype frequencies of 35% and 48.3% (p=0.582). No association between alcohol consumption and *GSTT1* and *GSTMI* null genotypes was found in these groups (p-values>0.05). There were more men, and alcohol consumption was prevalent in both groups.

**Conclusion:**

In this study we were unable to show a correlation between *GSTM1* and *GSTT1* genotypes and the development of head and neck squamous cell carcinomas in cigarette smokers.

## INTRODUCTION

Head and neck neoplasms are responsible for many deaths worldwide, being the sixth cause of death by câncer.^1^ The most frequent histological type is the squamous cell carcinoma, which is found in over 90% of cases,^2^, ^3^, ^4^ and is usually associated with the use of alcohol and tobacco.^5^, ^6^, ^7^, ^8^, ^9^ It is known that cigarette smoke is a complex mixture of over 4,000 substances, of which at least 40 are carcinogenic, initiating or promoting tumors in animals.^10^ Levels of electrophilic products launched into the bloodstream depend on the action of enzymes involved in biometabolism, which includes activation (Phase I) and detoxification (Phase II) of chemical compounds.^10^, ^11^ Polymorphisms in genes that codify these enzymes may alter their expression or function, altering carcinogenic compounds biometabolism^12^.

Various polymorphic genes that code enzymes involved in carcinogen biotransformation have been associated with cancer development.^13^, ^14^, ^15^, ^16^, ^17^, ^18^, ^19^, ^20^, ^21^, ^22^ Two genes in particular - *GSTT1* and *GSTM1* - that code phase II enzymes belonging to the glutathione S-transferases (GSTs) family seem relevant for susceptibility to head and neck squamous cell carcinoma; they detoxify carcinogenic tobacco smoke reactive metabolites.^11^, ^12^, ^13^, ^15^, ^18^, ^20^, ^23^

The *GSTM1* gene is polymorphic in humans, including a null-activity allele (*GSTM1*-) due to a major genic deletion, and two functional alleles (*GSTM1*A and *GSTM1*B). The *GSTT1* gene is also polymorphic in humans, and may have a deletion null genotype.^24^, ^25^ Individuals that have one of these null genotypes in homozygosis may be grouped into the negative conjugating phenotype, where there is complete loss of enzyme activity,^26^, ^27^ while those that have at least one functional allele are grouped in the positive conjugating phenotype.^28^

Thus, individual gene variability in the metabolic activation and detoxification process appears to be crucial to head and neck cancer susceptibility.

This study aimed to identify *GSTT1* and *GSTM1* gene null genotypes in smokers with head and neck squamous cell carcinoma and to compare these frequencies with those seen in smokers without a history of cancer, to identify possible susceptibility biomarkers for head and neck cancer.

## MATERIAL AND METHODS

Individuals diagnosed with squamous cell head and neck carcinomas confirmed by histopathology came from the Department of Otorhinolaryngology and Head and Neck Surgery of the Navy Hospital / Medical School of São José do Rio Preto and the Arnaldo Vieira de Carvalho Institute, SP. The control group included individuals with no history of neoplastic disease, paired by gender, age, ethnic group and use of alcoholic beverages. All subjects (patients and controls) were smokers. Individuals were included in the study after signing a free and informed consent form, and all information was obtained using a confidential standardized data-collection questionnaire (gender, ethnic group, smoking and use of alcohol). Information on smoking and alcohol-drinking was limited to a definition of user or non-user. The study was approved by the Research Ethics Committee of the Sao Jose do Rio Preto Medical School (CEP-FAMERP - 5639/2002) and the National Research Ethics Council (CONEP - legal process nº 842/2003).

Genomic DNA was extracted from peripheral blood according to the Abdel-Rahman et al technique.^29^ A sample of peripheral blood was collected in a test tube containing anticoagulant (EDTA) and lymphocytes were isolated using the Ficoll-Paque Plus. Genomic DNA was obtained by adding SDS (Sodium Dodecyl Sulphate), proteinase K and RNAse A to the isolated lymphocytes. Following NaCl purification, DNA was precipitated with ethanol and stored at -20ºC in a Tris-EDTA buffer solution for later analysis.

The analysis of *GSTT1* and *GSTM1* genes was simultaneously done by the chain reaction (PCR) according to Abdel-Rahman et al.^30^ Amplification of the DNA sequence of interest was obtained in 35 cycles that included DNA denaturing steps at 94ºC for 2 minutes, annealing of reaction-initiator sequences (primers) at 59ºC for 1 minute, and DNA chain extension by nucleotide addition at 72ºC for 1 minute. A *CYP1A1* gene exon 7 sequence was coamplified to serve as an internal amplification control. PCR products were analyzed in agarose gel at 1.5%, stained with ethydium bromide, and the null genotype (both alleles with a deletion) for the *GSTT1* and *GSTM1* genes was identified by the absence of amplification fragments of 480 base pairs (bp) and 219 bp respectively. Presence of the 312 bp fragment corresponded to the amplified *CYP1A1* gene sequence, which was evidence of a successful amplification reaction.

Demographics were presented as mean ± standard deviation (SD) or proportions. For the statistical analysis of the genotype frequencies obtained we used the exact Fisher test, with a significance level below 5%.

## RESULTS

Demography data: 120 individuals were recruited, of which 60 had head and neck squamous cell carcinoma (average age 54.6 ± 8 years) and 60 had no history of neoplastic disease (average age 54 ± 9 years). Men predominated (90% men vs. 10% women) and there were more alcohol drinkers (70% alcoholics vs. 30% non-alcoholics).

Primary sites: all head and neck squamous cell carcinoma cases were diagnosed and confirmed by pathology exams. The distribution of the primary tumor site is shown on Table 1. Mouth cancers were the most frequent type.

**Table 1:** Distribution of head and neck cancer cases by tumor site.

Site	Number (%)
Oral cavity	26 (43,3)
Supraglottic	15 (25)
Glottic	6 (10)
Subglottic	1 (1,7)
Pharynx	4 (6,7)
Hypopharynx	5 (8,3)
Other unknown primary sites	3 (5)

Frequency of polymorphisms: The *GSTT1* null genotype [-] was found in 33.3% (20 of 60) of patients and in 23.3% (14 of 60) of controls (P = 0.311). The *GSTM1* null genotype [-] was found in 21 (35%) of patients and 29 (48.3 %) of controls (p = 0.582). The combined *GSTT1* and *GSTM1* gene null genotype was seen in 10% (6 of 60) of patients and in 8.3% (5 of 60) of controls (p = 1.0). The most frequent genotypic combination, considering the presence of an unfavorable genotype (null GST), was *GSTT1* [+] / *GSTM1* [-] in 31.6% (19 of 60) of patients and 40% (24 of 60) of controls (p = 0.353) (Table 2).

**Table 2:** *GSTT1* and *GSTM1* genotype distribution in the experimental and control groups.

Genotypes*	Patients (n = 60) n (%)	Control (n=60) n (%)
Individual		

*GSTT1* [-]	20 (33,3)	14 (23,3)
*GSTM1*[-]	25 (41,7)	29 (48,3)

Combined *GSTT1*/*GSTM1*		

[-] / [-]	06 (10,0)	05 (8,3)
[+] / [+]	21 (35,0)	22 (36,7)
[+] / [-]	19 (31,6)	24 (40,0)
[-] / [+]	14 (23,3)	09 (15,0)

* Null genotypes [-], non-null [+].

Genotypes were grouped by tumor site (Table 3) and there was no correlation with *GSTT1* and *GSTM1* null genotypes.

**Table 3:** *GSTT1* and *GSTM1* genotype distribution by tumor site.

Genotypes	Oral Cavity	Supraglottic	Glottic	Subglottic	Pharynx	Hypopharynx	Others
*GSTT1* [+]	20	11	2	0	3	3	2
*GSTT1* [-]	6	4	4	1	1	2	1
*GSTM1* [+]	13	10	4	1	2	2	2
*GSTM1* [-]	13	5	2	0	2	3	1

^*^ Null genotypes [-], non-null [+].

Statistical analysis did not reveal any relation between alcohol use and *GSTT1* and *GSTM1* null genotypes [-] when we compared alcohol-drinking patients and controls (*GSTT1*, p = 0.34; *GSTM1*, p = 0.28; *GSTM1*[-] / *GSTT1*[-], p = 1.0).

## DISCUSSION

Epidemiological data has suggested that alcohol use and smoking are the main risk factors for malignant transformation in head and neck cancers.^31^, ^32^ It is difficult to assess the individual effect of these agents on the genesis of head and neck cancer. Smokers tend to consume alcohol and alcohol drinkers tend to smoke. Furthermore, in past decades the evidence has show that both factors operate synergistically, and as such may be the main cause of tumors.^32^

Studies have shown an association between alcohol drinking and the development of head and neck tumors when taking into account exposure time and the amount of alcohol consumed.^12^, ^34^ In our study, 70% of patients with cancer drink alcoholic beverages, which strengthens the association between alcohol use and the development of head and neck cancer. This is especially true in a sample that includes smokers. Unfortunately we were unable to obtain information about the amount of alcohol that was consumed or the exposure time.

The higher number of men in this study corroborates findings by Drummond et al,^22^ although our case number is not sufficient to explain this gender difference. An epidemiological study done at the A. C. Camargo Hospital in the state of Sao Paulo, Brazil, showed that the predominance of men in patients with mouth cancer appears to reflect differences in alcohol use and smoking between men and women in this country.^35^

The most frequent tumor site in our patients was the mouth, which is also the most frequent site for head and neck tumors reported in literature.^36^ This anatomical distribution may be explained by the fact that our sample was made up of smokers, most of which also consumed alcoholic beverages, reflecting the fact that the upper aerodigestive tract is more directly exposed to tobacco and alcohol.

*GSTT1* and *GSTM1* polymorphism studies done in Brazilian sub-populations reveal similar frequencies for both genotypes. Rossit et al,^37^ in a study among populations from the states of Para and Sao Paulo, showed frequencies of 18 and 47.3% for *GSTT1* and *GSTM1* gene null genotypes, respectively. Rossini et al,^38^ revealed frequencies of 25.4% for *GSTT1* [-] and 42.1% for *GSTM1* [-] in the state of Rio de Janeiro. Our study showed similar frequencies for these genotypes (24.4 and 17.8% for *GSTT1* [-] in patients and controls, and 44.4 and 48.9% for *GSTM1* [-] in patients and controls). Higher *GSTT1* [-] and *GSTM1* [-] polymorphism frequencies were observed by Drummond et al.^21^, ^22^ in Brazilian smokers with oral cavity squamous cell carcinoma (81.8% for *GSTT1* [-] and 70.5% for *GSTM1* [-]).

*GSTT1* and *GSTM1* polymorphism studies done in head and neck carcinomas are contradictory. Various authors have demonstrated an association with the *GSTM1* null genotype [-],^14^, ^15^, ^20^, ^21^, ^38^, ^40^, ^41^ which other authors have not seen.^5^, ^16^, ^42^, ^43^ A relation with the *GSTT1* null genotype [-] was also shown in some studies,^15^, ^20^, ^22^, ^44^ which was not seen in other studies.^5^, ^16^, ^37^, ^39^, ^41^, ^43^, ^46^ The combination of these higher risk and null genotypes has also been observed in this type of carcinoma.^14^, ^41^ Moreover, both exposure time and intensity to carcinogenic (alcohol and tobacco) may influence on the interaction of these genotypes for the development of this kind of neoplasia^1^, ^2^, ^34^, and these data were not considered in our study. A study by Konig-Greger et al^47^ showed that *GSTM1*-enzyme activity was significantly reduced in patients with head and neck carcinoma compared to controls, although it did not depend on the unfavorable *GSTM1* genotype, which may suggest that other enzymes participate as regulators.

Interestingly, Evans et al^13^ found that the positive *GSTT1* genotype [+] is associated with an increased risk of head and neck squamous cell carcinoma in smokers (OR = 1.6; CI 95% = 1.1-2.5) and that the *GSTT1* null genotype [-] may protect individuals against the development of this cancer. Although usually GSTs are considered detoxification enzymes, for certain specific chemical substrates such as dichloromethane (DCM), conjugation of glutathione with the GSTT1 enzyme may activate an electrophilic component, resulting in mutagenic potential.^48^, ^49^ Although DCM is not associated with tobacco, tobacco byproducts may gain carcinogenicity by GSTT1-enzyme mediated activation. Two other papers have also shown an increased risk of coronary disease and peripheral vascular disease in smokers with a GSTT1 genotype [+].50,51

## CONCLUSION

This study did not allow us to establish any correlation between *GSTT1* and *GSTM1* null genotypes and the development of head and neck squamous cell carcinoma in smokers.
